# Approximated Uncertainty Propagation of Correlated Independent Variables Using the Ordinary Least Squares Estimator

**DOI:** 10.3390/molecules29061248

**Published:** 2024-03-11

**Authors:** Jeong Sik Lim, Yong Doo Kim, Jin-Chun Woo

**Affiliations:** 1 Strategic Technology Research Institute, Korea Research Institute of Standards and Science (KRISS), Gajeong-ro 267, Yuseong-gu, Daejeon 34113, Republic of Korea; 2 Science of Measurement, University of Science and Technology (UST), Gajeong-ro 217, Yuseong-gu, Daejeon 34113, Republic of Korea; 3 Division of Chemical and Material Metrology, Korea Research Institute of Standard and Science (KRISS), Gajeong-ro 267, Yuseong-gu, Daejeon 34113, Republic of Koreajcwooo52@gmail.com (J.-C.W.)

**Keywords:** ordinary least squares, calibration, uncertainty evaluation, correlated independent variables, Monte Carlo simulation

## Abstract

For chemical measurements, calibration is typically conducted by regression analysis. In many cases, generalized approaches are required to account for a complex-structured variance–covariance matrix of (in)dependent variables. However, in the particular case of highly correlated independent variables, the ordinary least squares (OLS) method can play a rational role with an approximated propagation of uncertainties of the correlated independent variables into that of a calibrated value for a particular case in which standard deviation of fit residuals are close to the uncertainties along the ordinate of calibration data. This proposed method aids in bypassing an iterative solver for the minimization of the implicit form of the squared residuals. This further allows us to derive the explicit expression of budgeted uncertainties corresponding to a regression uncertainty, the measurement uncertainty of the calibration target, and correlated independent variables. Explicit analytical expressions for the calibrated value and associated uncertainties are given for straight-line and second-order polynomial fit models for the highly correlated independent variables.

## 1. Introduction

For calibration purposes, regression analysis wherein (in)dependent variables with a complex uncertainty structure has been intensively studied to yield the best estimates of the regression coefficient and associated uncertainties [1,2,3,4,5,6,7,8,9,10,11,12,13]. In particular, the uncertainty propagation of measured scalar responses toward the final calibrated value is a critical issue for ensuring the compatibility of inter- and intra-laboratory measurement results [14]. In the case of non-zero covariances among responses (dependent variables) and stimuli (independent variables), generalized least squares (GLS) regression is required to solve for the matrix form of squared residuals (SR), which is weighted against the variance–covariance matrix of calibration data vectors [15]. The weighted SR matrix is then minimized by an iterative solver. When the uncertainties of independent variables and covariances among dependent and independent variables are nonexistent, the GLS regression can be reduced to the weighted least squares (WLS) regression [1,2,3,4,5,6,7,8,16]. As the off-diagonal elements of the SR matrix are zero, the analytical expression for the fitted parameters of the regression model can be analytically derived.

In the metrological field, several research papers [17,18,19,20,21,22,23,24] and standards [25,26,27] have been published on regression analysis, and relevant softwares have been developed [28,29,30,31,32]. Analytical formulations of GLS are available from NPL and INRiM [18,20]. INRiM’s software, CCC (v2.0), works for linear and nonlinear regression problems with a full covariance matrix, excluding covariance between independent and dependent variables [28]. However, NPL’s software, XLGENLINE (v1.1), implemented in EXCEL by calling FORTRAN DLL, can work only for linear regression problems with up to fourth-order polynomials and diagonal covariance matrices [29]. In contrast, XGENLINE (v8.1) [30], which is a MATLAB version of XLGENLINE, uses a representation in terms of the Chebyshev polynomials to obtain better numerical conditioning than the general polynomials [17]. ISO/TS 28037:2010(E) [26] and the relevant software B_LEAST [30] can address the case of correlation among dependent and independent variables, but only when considering a straight-line model. B_LEAST can work with different types of regression models, including power and exponential models, but it requires diagonal covariance matrices only [32].

**Figure 1:**
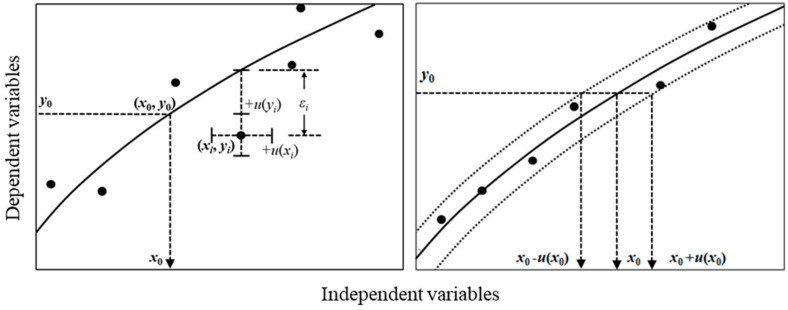
(**Left**) Notation of uncertainties and errors addressed in this study. *u*(*x_i_*) and *u*(*y_i_*) denote input variances in the reference and corresponding response values, respectively. The residual error *ε_i_* represents the deviation between its theoretical and input values along the ordinate. The measured response of the target is *y*_0_, which is calibrated to *x*_0_. (**Right**) Conceptual representation of linear dispersion of the calibrated value due to the fully correlated reference values. The tested dataset for the proposed calibration method is given in Table 1 in Section 2.5.

Following the Guide to the Expression of Uncertainty in Measurement (GUM), a common text for the metrological determination of the measurement value [33], the uncertainty of the calibration target can be evaluated by applying the (linear) law of propagation of uncertainty (LPU) to the abscissa. Therefore, for a generalized approach with the non-diagonalized SR matrix, an analytical process to derive the variance–covariance matrix of regression coefficients has been demonstrated only for particular cases wherein the correlation between variables does not disturb the derivation of the explicit variance–covariance formulae [33]. For chemical or biological RMs prepared from the same mother material, one may reasonably assume a strong correlation between these uncertainties [34]. For instance, a batch of chemical mixtures in various amount-fractions are typically blended using the same raw materials or mother mixture in the same facility and perhaps even by the same worker. Therefore, their variances may be correlated to some extent. Here, we attempt to derive an explicit expression of the uncertainty of the calibrated value which was propagated from measurement uncertainties of references and calibration targets using the ordinary least squares (OLS) estimator according to LPU. The calibration result using the proposed method is compared to the Monte Carlo simulation, complementing the validity of the proposed method. In addition, depicted in the right panel of Figure 1, a dataset in which the uncertainties of *y*_i_ are equivalent to the sum of squared residuals (SSR) and the uncertainties of *x*_i_ are highly correlated (Figure 1) was considered using LPU.

## 2. Theory

### 2.1. Least Squares Methods

Figure 1 illustrates the regression problem of the present study, presenting the measured data vector ***d***, *d_i_* = (*x_i_*, *y_i_*), where *y* corresponds to the response value and *x* the stimuli (typically, the value of the reference material (RM)), and reference values *x_i_* are fully correlated to each other. The minimization of regression variance for the data vector can be expressed as a generalized least squares (GLS) formulation as follows [13]:

(1) $${\left(\beta^{T},\;{X^{*}}^{T},{Y^{*}}^{T}\right)}^{T}=arg{min}_{x^{*},y^{*},\tilde{\beta }}{\left(\begin{array}{c} x-x^{*}\\ y-y^{*} \end{array}\right)}^{T}C_{d}^{-1}\left(\begin{array}{c} x-x^{*}\\ y-y^{*} \end{array}\right),$$

where the unobservable true vector describing the fitted line $\left(x^{*},y^{*}\right)$ is a constraint upon $x_{i}^{*}=q(y_{i}^{*},\tilde{\beta }$), *i* = 1, ∙∙∙∙, *n*. $\tilde{\beta }$ of order 1 × (*m* + 1) denotes the input regression coefficient vector of the *m*th-order polynomial model function, and $\left(\beta^{T},\;{X^{*}}^{T},{Y^{*}}^{T}\right)$ are auxiliary output quantities. ***C_d_*** is the variance–covariance matrix of the measurement data vector ***d*** of order *n* × *n*, where ***C_d_*** is assumed to have full rank. Equation (1) does not have a closed-form solution, and therefore, an iterative solving algorithm is required to derive the estimate $\hat{\beta }$ that is a vector of maximum likelihood estimators under the normality assumption, that is, the Best Linear Unbiased Estimator (BLUE) [17]. Equation (1) can be reduced to yield the OLS estimator at the regression coefficient vector ***β*** in the case that the ***C_d_*** is the identity matrix. The SSR at ***β*** is expressed as follows:

(2) $$S\left(\beta \right)={\left(y-X\beta \right)}^{T}(y-X\beta )$$

where $y={\left[y_{1}\;y_{2}\;\cdots \cdots \;y_{n}\right]}^{T}$, the design matrix ***X*** has *n* rows and *m* columns having elements of $x_{n}^{m}$, and y=Xβ+ε. The Gauss–Markov theorem allows the unobservable random error vector **ε** to have same variance *σ*^2^. The function ***S***(***b***) is quadratic in β with positive definite Hessian, and therefore, this function possesses a unique global minimum at β = $\hat{\beta }$**,** which can be given by the explicit expression as follows:

(3) $$\frac{\partial S}{\partial \beta }\left(\hat{\beta }\right)={\left.\frac{d}{d\beta }\left(y^{T}y-\beta^{T}X^{T}y-y^{T}X\beta +\beta^{T}X^{T}X\beta \right)\right|}_{\beta =\hat{\beta }}\;=-2X^{T}y+2X^{T}X\hat{\beta }=0$$

Therefore, a BLUE estimator of regression coefficients in the OLS problem can be derived from Equation (3) by applying the inverse of the Gram matrix ${\left(X^{T}X\right)}^{-1}$ to both sides. The measurement uncertainty (errors-in-variable on abscissa of data vector ***d***) can be considered identical to the standard deviation of fit residuals, and Equation (3) yields the OLS estimator that is the BLUE, satisfying the Gauss–Markov theorem as follows [35,36]:

(4) $${\hat{\beta }}_{OLS}={\left(X^{T}X\right)}^{-1}X^{T}y,$$

Therefore, ${\hat{\beta }}_{OLS}$ is an affine transformation of the response vector ***y*** onto a column space of the regression line by the orthogonal projection operator, ${\left(X^{T}X\right)}^{-1}X^{T}$ [37]. The GLS method is required when there are errors in the independent variable (along the ordinate). However, the error propagation of the regression variance of the GLS method requires, in general, the linearization of the regression variance at the solution point of Equation (1). The following sections explore the explicit expressions for the uncertainty of fully correlated reference *u*(*x_i_*) propagated via ${\hat{\beta }}_{OLS}$.

### 2.2. Variance–Covariance Matrix of the Regression Coefficient

A full matrix expression of Equation (4) can be expanded as follows:

(5) $${\left[\begin{array}{cc} \begin{array}{cc} n & \sum_{i=1}^{n}x_{i}\\ \sum_{i=1}^{n}x_{i} & \sum_{i=1}^{n}x_{i}^{2} \end{array} & \begin{array}{cc} \cdots \cdots & \sum_{i=1}^{n}x_{i}^{m}\\ \cdots \cdots & \sum_{i=1}^{n}x_{i}^{m+1} \end{array}\\ \begin{array}{cc} \begin{array}{c} \vdots \\ \vdots \end{array} & \begin{array}{c} \vdots \\ \vdots \end{array}\\ \sum_{i=1}^{n}x_{i}^{m} & \sum_{i=1}^{n}x_{i}^{m+1} \end{array} & \begin{array}{cc} \ddots & \begin{array}{c} \vdots \\ \vdots \end{array}\\ \cdots \cdots & \sum_{i=1}^{n}x_{i}^{2m} \end{array} \end{array}\right]}^{-1}\left[\begin{array}{c} \begin{array}{c} \sum_{i=1}^{n}y_{i}\\ \sum_{i=1}^{n}x_{i}y_{i} \end{array}\\ \begin{array}{c} \vdots \\ \vdots \end{array}\\ \sum_{i=1}^{n}x_{i}^{m}y_{i} \end{array}\right]-\left[\begin{array}{c} \begin{array}{c} \beta_{0}\\ \beta_{1} \end{array}\\ \begin{array}{c} \vdots \\ \vdots \end{array}\\ \beta_{m} \end{array}\right]=Q$$

Then, the cost function matrix ***Q*** is expressed as ***Q*** = (*q*_0_, *q*_1_, ……, *q_m_*)^T^ = ***0*** with coefficient vector ***β*** taken by the OLS estimator ${\hat{\beta }}_{OLS}$. Then, Equation (5) is reorganized to $X^{T}X\beta -X^{T}y=0$ to have the implicit function vector elements ***q_m_***, expressed as follows [38]:

(6a) $$q_{0}=\beta_{0}\cdot n+\beta_{1}\cdot \sum_{i=1}^{n}x_{i}+\cdots \cdots +\beta_{m}\cdot \sum_{i=1}^{n}x_{i}^{m}-\sum_{i=1}^{n}y_{i}=0$$

(6b) $$q_{1}=\beta_{0}\cdot \sum_{i=1}^{n}x_{i}+\beta_{1}\cdot \sum_{i=1}^{n}x_{i}^{2}+\cdots \cdots +\beta_{m}\cdot \sum_{i=1}^{n}x_{i}^{m+1}-\sum_{i=1}^{n}x_{i}y_{i}=0$$

(6c) $$q_{m}=\beta_{0}\cdot \sum_{i=1}^{n}x_{i}^{m}+\beta_{1}\cdot \sum_{i=1}^{n}x_{i}^{m+1}+\cdots \cdots +\beta_{m}\cdot \sum_{i=1}^{n}x_{i}^{2m}-\sum_{i=1}^{n}{x_{i}^{m}y}_{i}=0$$

Equation (6) does not require an estimation of the inverse matrix by organizing each element implicitly. The systematic equations of Equation (6) do not have an exact analytical solution. Instead, coefficient vector ***β*** fits the equation best in the sense of finding the minima of SSR as described above. According to DIN 1319-4, the uncertainty transformation matrix $T_{{\hat{\beta }}_{OLS}}$ for accounting for variability in ${\hat{\beta }}_{OLS}$ should be formed by the ordinate components of the regression coefficient vector ***β*** and the response vector ***y***. To propagate the regression variance, an uncertainty propagation matrix requires the Jacobian matrices of the cost function matrix ***Q*** against coefficient vector ***β*** as an input quantity and response vector ***y*** as an output quantity. Jacobian matrices ***Q****_i_* and ***Q****_o_*, where the subscripts represent the input and output quantities, respectively, yield an uncertainty transformation matrix $Q_{o}^{-1}Q_{i}$. To propagate the variances–covariances of the regression coefficients, the uncertainty propagation matrix requires Jacobian matrices of the cost function matrix ***Q*** against coefficient vector ***β*** and response vector ***y*** as input and output quantities, respectively. To obtain a positive definite, the uncertainty transformation matrix for estimating the OLS regression variance is negatively given as follows [23,39]:

(7) $$T_{{\hat{\beta }}_{OLS}}=-Q_{{\hat{\beta }}_{OLS}}^{-1}Q_{y},$$

where $Q_{y}$ and $Q_{{\hat{\beta }}_{OLS}}$ are the Jacobian matrices of the ***Q*** matrix against the response vector ***y*** and the regression coefficient vector β, respectively. These two matrices are given as follows:

(8) $$Q_{y}=\left[\begin{array}{cc} \begin{array}{cc} \frac{\partial q_{0}}{\partial y_{1}} & \frac{\partial q_{0}}{\partial y_{2}}\\ \frac{\partial q_{1}}{\partial y_{1}} & \frac{\partial q_{1}}{\partial y_{2}} \end{array} & \begin{array}{cc} \cdots \cdots & \frac{\partial q_{0}}{\partial y_{n}}\\ \cdots \cdots & \frac{\partial q_{1}}{\partial y_{n}} \end{array}\\ \begin{array}{cc} \begin{array}{c} \vdots \\ \vdots \end{array} & \begin{array}{c} \vdots \\ \vdots \end{array}\\ \frac{\partial q_{m}}{\partial y_{1}} & \frac{\partial q_{m}}{\partial y_{2}} \end{array} & \begin{array}{cc} \ddots & \begin{array}{c} \vdots \\ \vdots \end{array}\\ \cdots \cdots & \frac{\partial q_{m}}{\partial y_{n}} \end{array} \end{array}\right]=\left[\begin{array}{cc} \begin{array}{cc} -1 & -1\\ {-x}_{1} & {-x}_{2} \end{array} & \begin{array}{cc} \cdots \cdots & -1\\ \cdots \cdots & {-x}_{n} \end{array}\\ \begin{array}{cc} \begin{array}{c} \vdots \\ \vdots \end{array} & \begin{array}{c} \vdots \\ \vdots \end{array}\\ {-x}_{1}^{m} & {-x}_{2}^{m} \end{array} & \begin{array}{cc} \ddots & \begin{array}{c} \vdots \\ \vdots \end{array}\\ \cdots \cdots & {-x}_{n}^{m} \end{array} \end{array}\right]=-X^{T}$$

(9) $$Q_{{\hat{\beta }}_{OLS}}=\left[\begin{array}{cc} \begin{array}{cc} \frac{\partial q_{0}}{\partial \beta_{0}} & \frac{\partial q_{0}}{\partial \beta_{1}}\\ \frac{\partial q_{1}}{\partial \beta_{0}} & \frac{\partial q_{1}}{\partial \beta_{1}} \end{array} & \begin{array}{cc} \cdots \cdots & \frac{\partial q_{0}}{\partial \beta_{m}}\\ \cdots \cdots & \frac{\partial q_{1}}{\partial \beta_{m}} \end{array}\\ \begin{array}{cc} \begin{array}{c} \vdots \\ \vdots \end{array} & \begin{array}{c} \vdots \\ \vdots \end{array}\\ \frac{\partial q_{m}}{\partial \beta_{0}} & \frac{\partial q_{m}}{\partial \beta_{1}} \end{array} & \begin{array}{cc} \ddots & \begin{array}{c} \vdots \\ \vdots \end{array}\\ \cdots \cdots & \frac{\partial q_{m}}{\partial \beta_{m}} \end{array} \end{array}\right]=\left[\begin{array}{cc} \begin{array}{cc} n & \sum_{i=1}^{n}x_{i}\\ \sum_{i=1}^{n}x_{i} & \sum_{i=1}^{n}x_{i}^{2} \end{array} & \begin{array}{cc} \cdots \cdots & \sum_{i=1}^{n}x_{i}^{m}\\ \cdots \cdots & \sum_{i=1}^{n}x_{i}^{m+1} \end{array}\\ \begin{array}{cc} \begin{array}{c} \vdots \\ \vdots \end{array} & \begin{array}{c} \vdots \\ \vdots \end{array}\\ \sum_{i=1}^{n}x_{i}^{m} & \sum_{i=1}^{n}x_{i}^{m+1} \end{array} & \begin{array}{cc} \ddots & \begin{array}{c} \vdots \\ \vdots \end{array}\\ \cdots \cdots & \sum_{i=1}^{n}x_{i}^{2m} \end{array} \end{array}\right]=X^{T}X$$

The variance matrix of the regression coefficient $V_{{\hat{\beta }}_{OLS}}$ is calculated as follows:

(10) $$V_{{\hat{\beta }}_{OLS}}=T_{{\hat{\beta }}_{OLS}}\Omega T_{{\hat{\beta }}_{OLS}}^{T},$$

where ***Ω*** is the diagonal residual matrix with identical entries of *s*^2^ [2]. *s*^2^ is a variance of fit residual of the sampled data from the unobservable ‘true’ regression line, of which the regression coefficient vector is ***b***. Notably, the statistical parameter for describing the extent of dispersion of an unobservable error *e_i_* ~ *y_i_* − *q*(*x_i_*,***b***) that has a constant variance *σ*^2^, in which the error vectors are normally distributed $\varepsilon \sim N(0,\;\sigma^{2})$ (zero mean and standard deviation equaling *σ*). The standard deviation of the fit residuals of the sampled data, *s*, can be expressed by the following:

(11) $$s=\sqrt{\frac{\sum_{i=1}^{n}{\left\{y_{i}-\left(b_{0}+b_{1}x_{i}+\cdots +b_{m}x_{i}^{m}\right)\right\}}^{2}}{n-m-1},}$$

where the degrees-of-freedom correction in the denominator is restricted by the polynomial order of the fitting model *m* and the number of data vectors *n*. ${\hat{\beta }}_{OLS}$ allows the residual to be an observable estimate of the unobservable error ***ε***. The probability distribution of the residual error converges to a normal distribution following the Gauss–Markov assumption. The variance–covariance matrix of the OLS estimator $V_{{\hat{\beta }}_{OLS}}$ is expressed as follows:

(12) $$V_{{\hat{\beta }}_{OLS}}=s^{2}G^{-1},$$

where ***G*** is the Gram matrix $\left(X^{T}X\right)$ of Equation (9). Each diagonal entry corresponds to the variance of the regression coefficient of each term, whereas the off-diagonal entries correspond to the covariance between regression coefficients.

### 2.3. Propagation of Variance–Covariance of the Regression Coefficients

For calibration, the measured response value of the target *y*_0_ is inversely evaluated to obtain the corresponding stimulus value *x*_0_, which can be numerically rooted by using Newton’s method. To propagate the measurement uncertainty of *u*(*y*_0_) onto *u*(*x*_0_), the inverse evaluation requires the estimated regression coefficient vector ${\hat{\beta }}_{OLS}$ and estimated *x_0_* as input and output quantities, respectively. The Jacobian matrix against the input and output quantities ($q_{{\hat{\beta }}_{OLS}}$ and $q_{x}$, respectively) can then be expressed as follows:

(13) $$q_{{\hat{\beta }}_{OLS}}={\left[\frac{\partial q_{0}}{\partial \beta_{0}}\;\;\;\frac{\partial q_{0}}{\partial \beta_{1}}\;\;\;\cdots \cdots \;\;\frac{\partial q_{0}}{\partial \beta_{m}}\right]}_{x_{0}}=\left[1\;\;\;x_{0}\;\;\;\cdots \cdots \;\;x_{0}^{m}\right]$$

(14) $$q_{x}={\left[\frac{\partial q_{0}}{\partial x}\right]}_{x_{0}}=\beta_{1}+2\cdot \beta_{2}\cdot x_{0}+\cdots \cdots +{m\cdot \beta }_{m}\cdot x_{0}^{m-1}=q^{’}\left(x_{0}\right)$$

Here, $q^{’}\left(x_{0}\right)$ is the slope of the tangent of the determined regression line in the case of the second-order polynomial fit model at the target coordinate (*x*_0_, *y*_0_). This implies that the regression variance will be weighted by the slope of the tangent of the non-first-order calibration curve. The uncertainty transformation matrix for the regression variance toward the abscissa of the output quantity $t_{{\hat{\beta }}_{OLS}}$ is then given as follows:

(15) $$t_{{\hat{\beta }}_{OLS}}=q_{x}^{-1}q_{{\hat{\beta }}_{OLS}}$$

To transform the variance–covariance matrix of the OLS estimator $V_{{\hat{\beta }}_{OLS}}$ with the *m*th-order polynomial model function (Equation (12)) onto the abscissa corresponding to the output quantity *x_0_*, the uncertainty propagation matrix $t_{{\hat{\beta }}_{OLS}}$ (Equation (15)) is bracketed according to the LPU as follows:

(16) $$V_{m.{\hat{\beta }}_{OLS}}=t_{{\hat{\beta }}_{OLS}}V_{{\hat{\beta }}_{OLS}}t_{{\hat{\beta }}_{OLS}}^{T}$$

(17) $$\;\;\;\;\;\;\;\;={\left(\frac{s}{q^{’}(x_{0})}\right)}^{2}\left[1\;\;\;x\;\;\;x^{2}\;\cdots \cdots \;\;x^{m}\right]\left[\begin{array}{cc} \begin{array}{cc} g_{00} & g_{01}\\ g_{10} & g_{11} \end{array} & \begin{array}{cc} \cdots \cdots & g_{0m}\\ \cdots \cdots & g_{1m} \end{array}\\ \begin{array}{cc} \begin{array}{c} \vdots \\ \vdots \end{array} & \begin{array}{c} \vdots \\ \vdots \end{array}\\ g_{m0} & g_{m1} \end{array} & \begin{array}{cc} \ddots & \begin{array}{c} \vdots \\ \vdots \end{array}\\ \cdots \cdots & g_{mm} \end{array} \end{array}\right]{\left[1\;\;\;x\;\;\;x^{2}\;\cdots \cdots \;\;x^{m}\right]}^{T}$$

where *g_ij_* is an element of the inverse Gram matrix ***G***^−1^. In this study, $q^{’}\left(x_{0}\right)$ has only positive values. The propagated uncertainty of regression coefficients into the calibrated value *x_0_* is given as follows:

(18) $$u_{m}\left(x_{0}\right)=\frac{s}{q^{’}(x_{0})}\sqrt{\sum_{i=0}^{m}\sum_{j=0}^{m}g_{ij}\cdot x_{0}^{\left(i+j\right)}}$$

**Figure 2:**
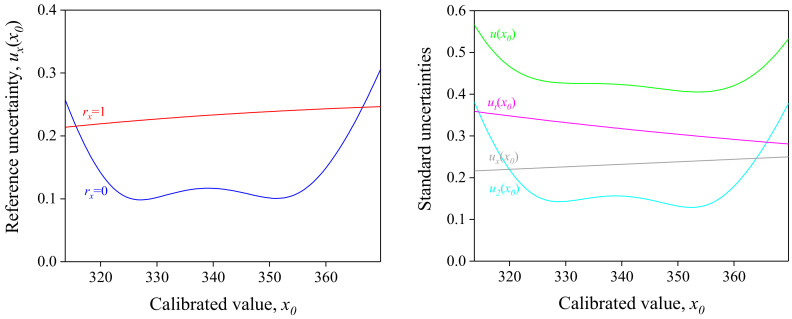
(**Left**) Propagated reference variance corresponds to the calibrated value when there is no correlation, *r_x_* = 0 (blue); and correlation is unity, *r_x_* = 1 (red). (**Right**) Uncertainties propagated from the uncertainties of reference *u_x_*(*x_0_*) (gray); target measurement, *u_t_*(*x_0_*) (magenta); and regression, *u_2_*(*x_0_*) (cyan). The combined standard uncertainty *u*(*x_0_*) is represented by the green line. For detailed discussions of *u_x_*(*x_0_*) and *u_t_*(*x_0_*), see Section 2.4 and Section 2.5.

Because the highest-order element of the inverse Gram matrix $g_{mm}$ is 2*m* order, for the second-order polynomial, *u*_2_(*x*_0_) is shown as a quartic curve, as shown in Figure 2. The variance of each component is estimated using the dataset in Table 1.

### 2.4. Propagation of the Measurement Uncertainty of the Calibration Target

To propagate the measurement uncertainty of the calibration target, its uncertainty value is treated as an input value of the reverse evaluation. The partial derivative of the cost function at *y_0_* can be expressed as follows:

(19) $$q_{y}=\left[\frac{\partial q_{0}}{\partial y_{0}}\right]=-1$$

Then, the uncertainty transformation matrix for the measurement uncertainty of the unknown sample can then be obtained to obtain the positive definite as follows:

(20) $$t_{t}=-q_{x}^{-1}q_{y}=\frac{1}{q^{’}\left(x_{0}\right)}$$

The propagation of the measurement uncertainty of the target *u*(*y_0_*) yields the target variance *V_t_* as follows:

(21) $$V_{t}=t_{t}u^{2}\left(y_{0}\right)t_{t}^{T}=\left[\frac{1}{q^{’}\left(x_{0}\right)}\cdot \left(\frac{s^{2}}{p}\right)\cdot \frac{1}{q^{’}\left(x_{0}\right)}\right],$$

where *s* is the measurement uncertainty of the calibration target, which, in general, can be approximated to the same extent of sampling variance for the regression data vector, and *p* is the number of repetitions of the target measurement. The measurement variance *s^2^* can be weighted by the number of repetitions *p* according to the central limit theorem. The target uncertainty can then be expressed as follows:

(22) $$u_{t}\left(x_{0}\right)=\frac{s}{q^{’}\left(x_{0}\right)}\sqrt{\frac{1}{p}}$$

### 2.5. Propagation of Uncertainty of the Independent Variable

The uncertainty of the independent variable was propagated using same method for the estimation of the regression variance. To define an uncertainty transformation matrix toward the abscissa, if there are no correlations between *x_i_* and *y_i_*, the Jacobian matrix of the cost function against the stimulus *x_i_* is defined as follows:

(23) $$Q_{x}=\left[\begin{array}{cc} \begin{array}{cc} \frac{\partial q_{0}}{\partial x_{1}} & \frac{\partial q_{0}}{\partial x_{2}}\\ \frac{\partial q_{1}}{\partial x_{1}} & \frac{\partial q_{1}}{\partial x_{2}} \end{array} & \begin{array}{cc} \cdots \cdots & \frac{\partial q_{0}}{\partial x_{n}}\\ \cdots \cdots & \frac{\partial q_{1}}{\partial x_{n}} \end{array}\\ \begin{array}{cc} \begin{array}{c} \vdots \\ \vdots \end{array} & \begin{array}{c} \vdots \\ \vdots \end{array}\\ \frac{\partial q_{m}}{\partial x_{1}} & \frac{\partial q_{m}}{\partial x_{2}} \end{array} & \begin{array}{cc} \ddots & \begin{array}{c} \vdots \\ \vdots \end{array}\\ \cdots \cdots & \frac{\partial q_{m}}{\partial x_{n}} \end{array} \end{array}\right]$$

The uncertainty transformation matrix of the reference uncertainty to the abscissa is given as follows:

(24) $$T_{x.{\hat{\beta }}_{OLS}}=Q_{{\hat{\beta }}_{OLS}}^{-1}Q_{x},$$

where $Q_{{\hat{\beta }}_{OLS}}$ is given in Equation (9). As an analytical derivation of the above matrix is nontrivial. Then, the variances and covariances of the references are propagated back to the abscissa using following equation:

(25) $$V_{x.{\hat{\beta }}_{OLS}}=T_{x.{\hat{\beta }}_{OLS}}V_{ref}T_{x.{\hat{\beta }}_{OLS}}^{T},$$

where the variance–covariance matrix of the reference $V_{ref}$ is given as follows:

(26) $$V_{ref}=D_{ref}R_{ref}D_{ref}$$

where $R_{ref}$ is the correlation matrix in which the elements are *r_x_*, and $D_{ref}$ is the diagonal matrix of the reference uncertainty. The uncertainty of the calibrated value of the target *u_x_*(*x_0_*) estimated from the OLS estimator is given as follows:

(27) $$u_{x}\left(x_{0}\right)=\frac{1}{q^{’}\left(x_{0}\right)}\sqrt{\sum_{i=0}^{m}\sum_{j=0}^{m}V_{ref}\left(i,j\right)\cdot {x_{0}}^{\left(i+j\right)}}$$

Unfortunately, the formulaic expression of the explicit form of *u_x_*(*x*_0_) is nontrivial for higher-order polynomials. In a condition of no correlation (*r_x_* = 0), a curvature of *u_x_*(*x_0_*) is quartically variated for the quadratic calibration function because of the nature of the regression variance–covariance matrix $V_{ref}$, of which the highest-order element is $x^{2m}$. (Figure 2).

**Figure 3:**
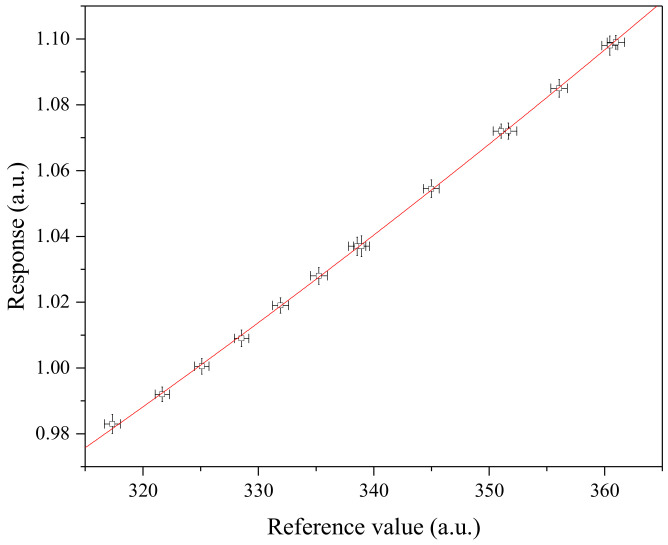
Graphical representation of the artificial dataset A given in Table 1. Note that the uncertainties of reference values and responses are amplified three times to be seen. The uncertainties of reference values were assumed to be fully correlated with each other.

As a quantitative investigation of the correlation coefficient is beyond the limitation of the realization in many cases, a conservative approach is required. In an extremely conservative case, for an all-ones correlation matrix (all $r_{x_{ij}}=1$), the measurement uncertainty of the target along the abscissa is coherently dispersed, as presented below:

(28) $$u_{x}\left(x_{0}\right)\sim \frac{1}{2}\left|\begin{array}{c} q\left(x_{1}+u\left(x\right),\;x_{2}+u\left(x\right),\;\cdots \cdots ,\;{\;x}_{n}+u\left(x\right),\;{\;y}_{1},\;y_{2},\;\cdots \cdots \;\;y_{n},\;{\;y}_{0}\right)\\ \;-q\left(x_{1}+u\left(x\right),\;x_{2}+u\left(x\right),\;\cdots \cdots ,\;{\;x}_{n}+u\left(x\right),\;{\;y}_{1},\;y_{2},\;\cdots \cdots \;\;y_{n},\;{\;y}_{0}\right) \end{array}\right|,$$

where *q* is the cost function of the fitted model of the OLS estimator and *u*(*x*) is the averaged uncertainty of *u*(*x_i_*). It was assumed that the uncertainty values of references are close to each other, as shown in Table 1 and Figure 3. The fitted line spans out in the positive and negative directions by the extent of the width of the normal distribution, of which uncertainty is *u*(*x*) (Figure 1). As expected, *u_x_*(*x_0_*) is smoothly variated as a function of *x_0_* when all correlations are fully positive (i.e., *r_x_* = 1) based on the assumption that *u*(*x_0_*) is constant with respect to *x_0_* (Figure 2). The variation rate is lower at large values of *x_0_* because the slope of the tangent for the used dataset is higher at such values. In the case that *x*_0_ is centered within the reference scale, Equation (26) can be simplified as follows:

(29) $$u_{x}\left(x_{0}\right)≅\bar{u_{ref}}$$

**Table 1:** Datasets to test the proposed method in this study. Note that all *u*(*y_i_*) values are close to the standard deviation of the fit residual of the corresponding dataset. The uncertainties of reference values were assumed to be fully correlated with each other.

Reference (*x_i_*)	*u* (*x_i_*)	Response	*u*(*y_i_*)		
			Set A	Set B	Set C
317.35	0.231	0.983	0.00098	0.00098	
321.68	0.206	0.992	0.00074	0.00074	0.00041
325.11	0.208	1.001	0.00080		
328.54	0.209	1.009	0.00085	0.00085	
331.92	0.233	1.019	0.00077	0.00077	0.00042
335.24	0.243	1.028	0.00085		
338.56	0.252	1.037	0.00093	0.00093	
338.93	0.231	1.037	0.00106	0.00106	0.00058
344.98	0.230	1.055	0.00089		
351.03	0.229	1.072	0.00072	0.00072	
351.66	0.246	1.072	0.00082	0.00082	0.00045
356.06	0.239	1.085	0.00090		
360.45	0.232	1.098	0.00098	0.00098	
360.97	0.253	1.099	0.00069	0.00069	0.00038
364.85	0.241	1.111	0.00085		
Target	(*p* = 1)	1.003	*n* = 15	*n* = 10	*n* = 5

## 3. Discussions

Artificial datasets mimicking the measurements of ambient-level N_2_O using a gas chromatograph with an electron capture detector (GC-ECD) were utilized to evaluate the uncertainty of calibrated value *x_0_* (Table 1). The artificial data are dispersed along the ordinate to have uncertainties, *u*(*y_i_*), which are closely set to the standard deviation of fit residuals *s* = 0.00073, 0.00085, and 0.00041, respectively, for dataset A, B, and C. This aspect implies that *u*(*y_i_*) are considered as the sampling uncertainties from the $\left(x^{*},y^{*}\right)$ which was modeled by the OLS estimator. It should be noted that the OLS and the WLS methods yield similar results in the regression variance, where the variance–covariance matrix of the WLS estimator $V_{{\hat{\beta }}_{WLS}}$ was computed by the equation of ${\left(X^{T}WX\right)}^{-1}$**,** where *W* is the weighting matrix and its elements are inversed *u*(*y_i_*) [5]. The *u_x_*(*x*_0_) curves for *r_x_* = 1 and *r_x_* = 0 cross outside of the range of the reference scale, implying that a conservative approach can be achieved by considering the correlation effect of reference values within the reference scale (Figure 2). The post-evaluation of the correlation factors among reference values is hard to achieve; one may assume *r_x_* = 1 to avoid underestimating the calibration uncertainty due to uncertainties of reference values.

**Figure 4:**
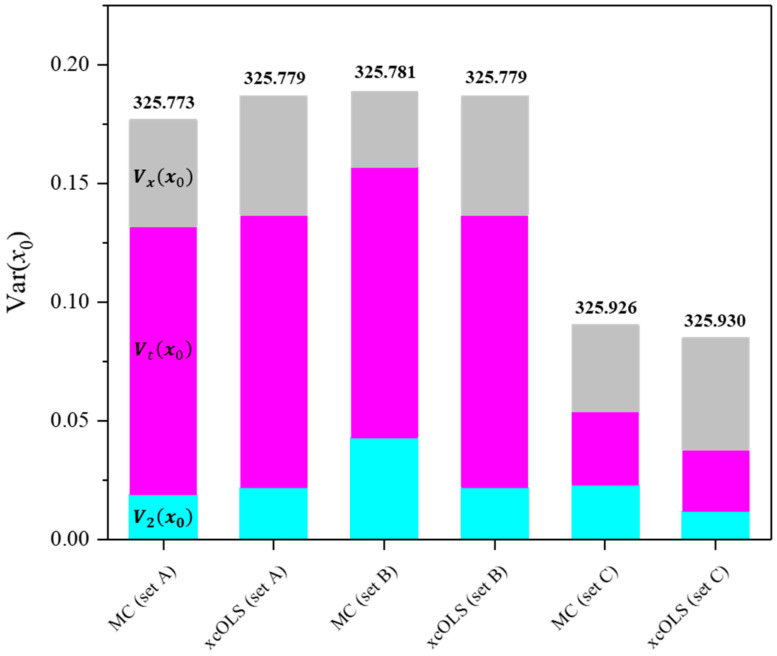
A comparison of the variance values corresponding to the reference, $V_{x}\left(x_{0}\right)$ (gray); the target measurement, $V_{t}\left(x_{0}\right)$ (magenta); and regression, $V_{2}\left(x_{0}\right)$ (cyan). Note that the number of target measurement repetitions *p* is 1. Calibrated values obtained through each method are indicated on top of the respective bars.

To verify the derived explicit equation of each uncertainty value, the Monte Carlo (MC) simulation was conducted employing identical datasets. In the MC method, individual data points were randomly sampled from normally distributed reference values (*x_i_*), which were fully correlated to each other (all $r_{x_{ij}}=1$), and normally distributed response values (*y_i_*). The uncertainties of both *x_i_* and *y_i_*, denoted as *u*(*x_i_*) and *u*(*y_i_*), respectively, for each dataset are detailed in Table 1. A total of 10,500 ***d****_i_* = (*x_i_*, *y_i_*) were randomly sampled from the population to compute the uncertainty value of the calibration target. Running the OLS estimator on each sampled data vector yielded the single regression coefficient vector ***β*** and calibrated value *x*_0_. An average and standard deviation of each parameter resulted in the corresponding value and its uncertainty. Because it is a valid assumption that a result by the MC simulation will be in close proximity to the unbiased limit of variance of the resulting distribution [40], a comparison between the *x*-correlated OLS method (*_xc_*OLS) and the MC method allowed us to test the reliability of the *_xc_*OLS method (Figure 4). With LPU, it is hard to handle the GLS problem in which the SR matrix cannot be reduced to the implicit model equations [33]. Therefore, approximating the uncertainties according to the LPU is prevalent in many cases. The uncertainties *u*_2_(*x*_0_), *u_t_*(*x*_0_), and *u_x_*(*x*_0_) from both the *_xc_*OLS and MC methods exhibit a high degree of agreement for various datasets in ranges of variance of fit residuals *s*^2^. Covariances stemming from the coherent dispersion of reference values were observed to be minor, as seen in the comparison between the MC and *_xc_*OLS methods in the datasets (Table 2 and Table 3 and Figure 4).

## 4. Conclusions

In the field of chemical and biological analysis, the OLS method is a prevalent choice for calibration tasks involving highly accurate RMs, particularly when the negligible uncertainty of references obviates the need for their consideration in the regression analysis. However, numerous scenarios, such as those encountered in graphite furnace atomic absorption spectroscopy, X-ray fluorescence spectroscopy, and spark emission spectroscopy, deviate from the simplicity of the OLS approach due to the presence of considerable variance–covariance in the independent variables, namely reference values. To address this challenge, an approximated propagation method for the variance–covariance of the data matrix becomes imperative. This study addresses a specific calibration scenario wherein the independent variables exhibit high correlation, and uncertainties along the ordinate are equivalent to the standard deviation of the fit residuals. If the uncertainties of the independent variables are deemed negligible, this scenario aligns with the traditional OLS case. Conceptually illustrated in Figure 1, the uncertainties of fully correlated independent variables were dispersed orthogonally along the abscissa. The validity of this concept is substantiated through the step-by-step derivation of explicit equations. This study meticulously budgets the contributions of variances arising from regression coefficients, target measurements, and references, offering an analytical framework for comprehending uncertainty propagation for the calibration scenario. The calibration task employing the *_xc_*OLS and the MC methods was exemplified using artificial datasets, with standard deviations of fit residuals *s* being 0.00073, 0.00085, and 0.00041. The resulting uncertainty values, as a metric for comparing results, were estimated by the *_xc_*OLS and the MC methods to show good agreement. This good agreement underscores the validity of the proposed approach in propagating uncertainties associated with highly correlated independent variables using the OLS estimator.

## Figures and Tables

**Table 2:** Estimated regression coefficients for each dataset. Associated uncertainties are given in the parentheses.

Dataset	Method	Regression Coefficients (Uncertainty)		
		*β*_0_	*β*_1_	*β*_2_
A	MC	0.7643 (0.1441)	−0.001084 (0.000844)	0.0000056 (0.0000012)
	*_xc_*OLS	0.7642 (0.1445)	−0.001084 (0.000846)	0.0000056 (0.0000012)
B	MC	0.8084 (0.2368)	−0.001338 (0.001394)	0.0000059 (0.0000020)
	*_xc_*OLS	0.8080 (0.1724)	−0.001336 (0.001015)	0.0000059 (0.0000015)
C	MC	0.6767 (0.1866)	−0.000565 (0.001089)	0.0000048 (0.0000016)
	*_xc_*OLS	0.6785 (0.1355)	−0.000576 (0.000794)	0.0000048 (0.0000012)

**Table 3:** The combined standard uncertainty of the determined value of the calibration target under the assumption of unitive correlation of the reference values. *β*_1_ is the slope of a fitted straight line, and $\bar{x}$ is the mean of the reference value. The measurement uncertainty of the target is assumed to be at a level similar to the measurement uncertainty of the reference. The unitive correlation among the reference variances is approximated to $u_{x}\left(x_{0}\right)\;≅\;\bar{u_{ref}}$.

Polynomial Order	$Var(x_{0})$
*m* > l	$\frac{s^{2}}{{\left(q^{’}\left(x_{0}\right)\right)}^{2}}\left(\frac{1}{p}+\sum_{i=0}^{m}\sum_{j=0}^{m}g_{ij}\cdot x^{\left(i+j\right)}\right)+{\bar{u_{ref}}}^{2}$
*m* = 1	$\frac{s^{2}}{\beta_{1}^{2}}\left(\frac{1}{p}+\frac{1}{n}+\frac{{\left(x_{0}-\bar{x}\right)}^{2}}{\sum_{i=1}^{n}{\left(x_{i}-\bar{x}\right)}^{2}}\right)+{\bar{u_{ref}}}^{2}$

## Data Availability

Data are contained within the article.
